# Cytokine-induced killer cells mediated pathways in the treatment of colorectal cancer

**DOI:** 10.1186/s12964-022-00836-0

**Published:** 2022-03-28

**Authors:** Farimah Fayyaz, Niloufar Yazdanpanah, Nima Rezaei

**Affiliations:** 1 Students’ Research Committee, Alborz University of Medical Sciences, Alborz, Iran; 2 Network of Immunity in Infection, Malignancy and Autoimmunity (NIIMA), Universal Scientific Education and Research Network (USERN), Alborz, Iran; 3 Research Center for Immunodeficiencies, Children’s Medical Center Hospital, Tehran University of Medical, Dr. Qarib St, Keshavarz Blvd, Tehran, 14194 Iran; 4 Network of Immunity in Infection, Malignancy and Autoimmunity (NIIMA), Universal Scientific Education and Research Network (USERN), Tehran, Iran; 5 School of Medicine, Tehran University of Medical Sciences, Tehran, Iran; 6 Department of Immunology, School of Medicine, Tehran University of Medical Sciences, Tehran, Iran

**Keywords:** Cytokine-induced killer cell, Colorectal cancer, Immunotherapy, Treatment, Solid tumors

## Abstract

Cytokine-induced killer (CIK) cell therapy is a type of adoptive immunotherapy that due to its high proliferation rate and anti-tumor characteristics, is being investigated to treat various solid tumors. Since advanced colorectal cancer (CRC) has high mortality and poor survival rates, and the efficacy of chemotherapy and radiotherapy is limited in treatment, the application of CIK cell therapy in CRC has been evaluated in numerous studies. This review aims to summarize the clinical studies that investigated the safety and clinical efficacy of CIK cell therapy in CRC. Therefore, 1,969 enrolled CRC patients in the clinical trials, of which 842 patients received CIK cells in combination with chemotherapy with or without dendritic cell (DC) infusions, were included in the present review. Furthermore, the signaling pathways involved in CIK cell therapy and novel methods for improving migration abilities are discussed.

**Video abstract**

**Supplementary Information:**

The online version contains supplementary material available at 10.1186/s12964-022-00836-0.

## Background

Colorectal cancer (CRC), as the second leading cause of cancer death in both men and women, ranks third among malignancies in terms of incidence [1]. Approximately 104,270 new cases and 52,980 deaths due to CRC were estimated to occur in the United States in 2021 [2]. The incidence of CRC is increasing in younger populations and countries with medium to high Human Development Index (HDI) [3]. Surgical resection, chemotherapy, and radiotherapy are the main treatment options. However, chemotherapy carries an increased risk of severe side effects [4]. Currently, the 5-year survival rate for CRC patients is estimated to be about 64%. Albeit, it is highly influenced by the stage of cancer at diagnosis. It ranges from 90% for patients diagnosed in early phases with the localized disease to 14% for patients diagnosed with distant metastasis [5]. The novel systemic therapies in metastatic CRC patients, including biologic agents and immune checkpoint inhibitors, have improved the clinical outcomes [6].

Adoptive immunotherapy is a novel approach for treating different types of cancer. Cytokine-induced killer (CIK) cells are major histocompatibility complex (MHC)-unrestricted anti-tumor effector CD3 + T lymphocytes that are easily generated by ex vivo expansion of peripheral blood mononuclear cells (PBMCs) with anti-CD3 antibodies, interleukin-2 (IL-2), and interferon-gamma (IFN-γ) [7, 8]. The cytotoxicity of CIK cells depends on the engagement of natural killer group 2 member D (NKG2D) with its ligand on tumor cells and the progress of perforin-mediated pathways [9]. CIK cells have a higher proliferation rate and more potent anti-tumor activity than lymphokine-activated killer (LAK) cells and tumor-infiltrating lymphocytes (TILs), and they have considerable potential in the treatment of solid tumors. CIK cells have been tested on various types of solid tumors and hematologic malignancies [10].

CIK cell therapy in CRC patients is under investigation, and despite its promising results, it is not a commonly applied approach in CRC. The present article summarizes the reported mechanisms and pathways mediated by CIK cell therapy in CRC and clarifies the novel methods used in CIK cell therapy procedures. Moreover, the outcomes of CIK cell clinical trials on CRC patients will be reviewed to determine the advantages and disadvantages of this course of treatment.

## The phenotype of CIK cells

The phenotypic characteristics of generated CIK cells and their ratio are heterogeneous from patient to patient [11]. The majority of studies on the CIK phenotype of CRC patients showed a significant increase in the percentages of CD3+, CD8+, CD3+CD56+, and CD3+CD4+ subsets after culture for CIK therapy [12–16]. CIK cells express NKG2D, CD56 DNAX accessory molecule-1 (DNAM-1), and NKp30, which are essential in cytotoxicity against tumor cells [17, 18]. Additionally, CD8 and LCK proto-oncogene, a member of Src family tyrosine kinase, is also attributed to the cytotoxicity of CIK cells [19]. Moreover, the dynamic analysis of immune checkpoints on CIK cells of non-small cell lung carcinoma (NSCLC) patients demonstrated that the expression of immune checkpoints increased during the early stages of the culture. Although PD-L1, LAG-3, TIM-3, and CEACAM-1 remained at a high level during CIK culture, the expression of TIGIT, BTLA, PD-1, and CTLA-4 decreased gradually [20]. Similarly, in patients with hematologic malignancies, the expressions of LAG-3 and TIM-3 were present in CIK cultures; meanwhile, CTLA-4 and PD-1 expressions were low [21].

The variation in the improvement of immune function among patients receiving CIK therapy could alter their clinical response. In a study by Pan et al*.*, 42 CRC patients were treated with post-surgical CIK cell therapy. The median ratios of CD3+CD4+, CD3+CD8+, CD3+CD56+, and CD3–CD56+ subgroups were 27.36%, 60.53%, 10.63%, and 3.76%, respectively. The analysis of the association of CIK phenotype to the overall survival (OS) demonstrated that a high ratio of the CD3+CD4+ subset was associated with poorer OS. On the other hand, a high ratio of the CD3+CD8+ subset was associated with better OS in CRC patients. However, the results were not significant [22].

The phenotypes of CIK cells of 51 CRC patients who were treated with CIK therapy were analyzed in a more recent study from Pan et al*.*, in which the median ratio of CD3+, CD3+CD4+, CD3+CD8+, CD3–CD56+, and CD3+CD56+ subgroups were similar to the previous study and were 97.5%, 29.2%, 66.3%, 1.9%, and 16.4%, respectively, among which the percentages of CD3+CD56+ and CD3+CD8+ subsets were significantly higher than the peripheral blood mononuclear cells (PBMCs). In addition, the rate of the CD3–CD56+ subset was significantly lower than the PBMCs. The assessment of the phenotypic evolution of CIK cells through the first four cycles showed that the CD3+CD4+ subset ratio significantly decreased. At the same time, the proportions of CD3–CD56+ and CD3+CD56+ significantly increased after the fourth cycle [23]. Since the CD3+CD56+ subset is known as the essential anti-tumor immune cells and the rate of it increases after culture for CIK therapy [24], Pan et al*.* evaluated the relationship between the percentage of CD3+CD56+ subset in CIK cells and survival of treated metastatic CRC patients and showed that no significant differences were observed in the OS and progression-free survival (PFS) in higher ratios of CD3+CD56+ subset in the first cycle. However, after the fourth cycle, the increase in the CD3+CD56+ subset was associated with improved OS and PFS [25].

## Modification of tumor-targeted migration of CIK cells

Even though CIK therapy is reported to be beneficial in various clinical trials, given the requirement of a large number of CIK cells transfusions, there is a need to improve the CIK cell activation towards the autologous cancer cells.

In 2012, CEA-specific CAR-CIK cells were designed to express either CD3γ or CD28-CD3γ signaling domains. They were co-incubated with CEA^+^ Colo205 and CEA^−^ Colo201 CRC cells, and the secretion of IFN-γ was assessed to evaluate their tumor specificity. The IFN-γ secretion was increased in the CAR-CIK cells co-incubation with CEA^+^ CRC cells. Indeed, the increase was more significant in the CIK cells activated by the CD28-CD3γ rather than CD3γ CAR-CIK cells. Moreover, the evaluation of CEA-specific CAR-CIK cells in CRC cells isolated from surgical specimens confirmed the same results [26].

Since chemokines could mediate the migration of immune cells to tumors by corresponding with chemokine receptors, Zou et al*.* investigated the chemokine expression profiles in CRC cells and chemokine receptor expression profiles on CIK cells derived from the same donors. They demonstrated that the CXCR3 and CXCR4 were expressed in higher levels on the CIK cells of CRC patients compared with healthy controls. Moreover, the examination of chemokine expression profiles of CRC tissues and cell lines showed the overexpression of CXCL10, CXCL11, and CCL3 compared with the adjacent normal samples. Another significant observation of the team was that expression levels of chemokine receptors on CIK cells decreased during the expansion process. To increase the tumor-targeted migration, they re-treated CIK cells with CXCL11 on day seven and CCL21 on day 14, which boosted the CIK migration ability evaluated by Transwell assay [27]. They had also previously re-stimulated CIK cells with anti-CD3/anti-CD28-coated beads that increased chemokine receptor expression of CIK cells and their cytolytic ability [28].

## Signaling pathways involved in CIK Cell therapy

### NKG2D/NKG2DL

The MHC-unrestricted cytotoxicity of CIK cells is mainly attributed to the interaction of NKG2D, a type 2 transmembrane protein, with its ligands compromising of MHC class I-like molecules and MHC class I-related genes, which include MHC class I-related genes A and B (MICA and MICB) and UL16-binding protein (ULBP). The expression of NKG2D and its associated adaptor molecule, DAP-10, is upregulated in CIK cells [9]. The NKG2D ligands (NKG2DLs) expressions on tumor cells play an essential role in recognizing and eliminating tumor cells by NKG2D + effector cells [29]. Genomic and cellular stress induces the expression of NKG2DLs, which is mainly confined to tumor cells [30].

Several studies have investigated the cell surface expression of NKG2DLs in CRC. In a study in 2006, 449 CRC tumors were examined for the expression of MICA. The results showed that the expression of MICA was present in more than 75% of tumor cells of all studied CRC tumors [31]. Next, the expressions of MIC, ULBP1, ULBP2, and ULBP3 were assessed in 462 primary CRC tumors. The majority of CRC tumors expressed the NKG2DLs. The expression level of NKG2DLs was associated with prognosis, whereby a high level of MIC expression was correlated with improved survival, and the expression level of all NKG2DLs reduced with the increase in tumor stage [32]. Feng et al*.* published the results of their prospective cohort study on the MICB expression in CRC and its association with prognosis in 2020. It was demonstrated that there was a significant positive association between high MICB expression and OS of CRC patients [33].

Moreover, Kucuk et al*.* undertook the analysis of NKG2DLs expressions in response to low doses of bortezomib and epirubicin, demonstrating that MICA expression slightly increased in CRC cell lines [34]. Another recent study evaluated MICA expression in 192 tumor, and adjacent normal tissue samples from 96 CRC patients revealed that MICA expression was significantly higher in CRC tumors than the adjacent normal tissue. In contrary to previous results, the overexpression of MICA was associated with poor prognosis [35].

### AMPK/Akt/mTOR

AMP-activated protein kinase (AMPK) is a serine/threonine-protein kinase complex activated by various external stimuli through cellular energy depletion. It consists of a catalytic α-subunit, a regulatory β- and γ-subunits [36]. Phosphorylated AMPK can promote apoptosis and suppress the proliferation of tumor cells by inhibiting the mammalian target of rapamycin (mTOR) pathway [37, 38]. It has been previously demonstrated that mTOR signaling activation occurs in CRC tumorigenesis [39]. FOXM1, a member of the Forkhead transcription factors family, which is regulated by FOXO transcription factors, has a role in tumorigenesis and cancer progression. The overexpression of FOXM1 is observed in various cancers, including CRC [40–42]. Akt, a serine/threonine kinase, can activate mTOR through PI3K/Akt/mTOR pathway [43]. Additionally, activated Akt has a role in the regulation of FOXO transcription factors [44].

**Fig. 1:**
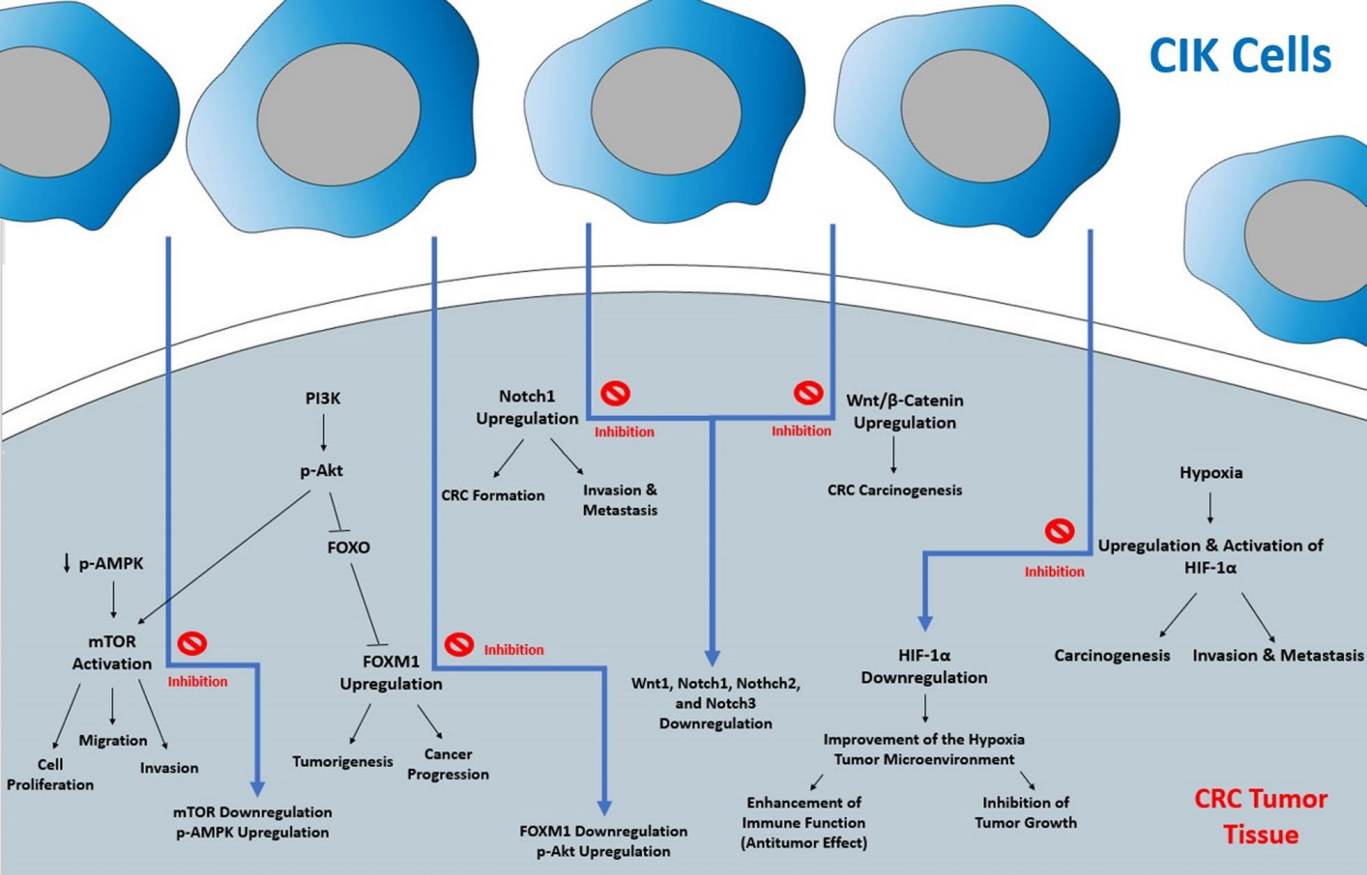
The effect of CIK cell therapy on AMPK/Akt/mTOR, WnT/β-Catenin, and HIF-1α signaling pathways in colorectal carcinoma

In a study by Shi et al*.*, human CRC cell line SW480 was co-cultured with CIK cells. The AMPK/Akt/mTOR pathway evaluation indicated that p-AMPK and p-Akt were upregulated, while FoxM1 and p-mTOR were down-regulated compared to that of the SW480 cells alone. A CCK-8 assay was used to investigate the proliferation ability of CIK cells, which demonstrated that CIK cells significantly inhibited the proliferation of SC480 cells (*P* < 0.05). Moreover, Hoechst staining was performed to assess the effect of CIK cells on the regulation of apoptosis. It was indicated that the apoptosis rate of SW480 cells co-cultured with CIK cells was significantly higher than that of SW480 cells alone (*P* < 0.05). Furthermore, the number of invasive cells in the CIK + SW480 cells was significantly lower than that of SW480 cells (*P* < 0.05) [45]. This showed the anti-tumor effect of CIK cells in proliferation, apoptosis, the number of invasive cells in CRC, and the possible role of the AMPK/Akt/mTOR pathway (Fig. 1).

### Notch and Wnt/β-catenin

The notch signaling pathway regulates cell growth, differentiation, and apoptosis and has a crucial role in different types of cancer [46]. It was previously reported that upregulation of Notch1 expression may affect CRC formation and is associated with a poor OS rate in CRC [47, 48]. In addition, the Wnt/β-Catenin signaling pathway, which controls cell growth, migration, and embryogenesis, has an essential role in intestinal homeostasis, and its dysregulation could contribute to CRC [49, 50].

In an animal study, Akt, Wnt1, Notch1, Nothch2, and Notch3 mRNA expressions after treatment with DC-CIK, Huaier Granule, or both combined in HT-29 colon cancer xenograft mice showed to be significantly downregulated as compared with the blank control (*P* < 0.05) [51] (Fig. 1).

### HIF-1α

Hypoxia-inducible factor-1α (HIF-1α) is a transcription factor that has an essential role in cellular reaction to oxygen stress state, as hypoxia stabilizes and activates the factor [52]. It has been reported that HIF-1α is activated in many solid tumors, including CRC, and is associated with the progression of tumors [53, 54].

To assess the effect of CIK cells on HIF-1α, colon 26 cancer xenograft mice were divided into the CIK, normal saline (NS), and control groups. It was indicated that the HIF-1α expression was significantly decreased in the CIK group's tumor tissue compared to that of the other groups (*P* < 0.05). Moreover, the results confirmed that the expression of HIF-1α was significantly higher in the tumor tissue in comparison with the small intestine (*P* < 0.05) [55] (Fig. 1).

## Role of CIK cell in gene therapy

The inhibition of tumor progression could be achieved by transferring specific genes to CRC cells to suppress the function of defective genes involved in CRC development [56]. One of the methods used in transferring genes to cells is the application of viral vectors. Adenoviral vectors are the primary vectors used in CRC gene therapy and could be highly produced [57]. However, the delivery of adenovirus to the target tumor is not optimal. Therefore, Liu et al*.* aimed to improve the delivery by using CIK cells as second vectors. P21Ras could be a potential target for gene therapy in CRC. Liu et al*.* prepared adenovirus KGHV500 carrying anti-p21scFv to assess its ability to penetrate tumor cells and inhibit cell growth. Since CIK cells express CD46 marker, a KGHV500 receptor, they co-cultured the recombinant adenovirus with CIK cells and injected it into SW480 CRC cell xenograft mice to investigate its inhibitory effect in vivo in comparison with CIK therapy or recombinant virus injection alone. The results elucidated that the suppression of tumor volume and the apoptosis rate were significantly higher in KGHV500 combined with the CIK cells group compared to the other treatment groups. Moreover, the immunohistochemistry of xenograft tumors and other organs demonstrated that in the KGHV500 combined with CIK cells group, the number of observed KGHV500 viruses in the tumor was higher than in other groups, while no virus was detected in the heart, liver, or lungs. The expression of scFv was higher in the KGHV500 combined with the CIK cells group, and scFv was only expressed in the tumor and spleen in this group, while in the KGHV500 group, it was expressed in all tissues. The study results favored the safety and efficacy of the delivery [58]. Similar in vitro and in vivo results with the administration of the co-culture of CIK cells and recombinant oncolytic adenovirus KGHV500 carrying anti-p21Ras scFv were found in lung cancer, glioma, and liver cancer [59–61].

E2F1 is a transcription factor that could induce cells to transit from the G0/G1 phases to the S phase of the cell cycle. Due to the overexpression of E2F1 in tumor tissue, it could be a potential target for gene therapy. It was demonstrated that the adenovirus-E2F1 promoter regulator has therapeutic efficacy in rectal cancer. To evaluate the synergistic anti-tumor effect of this recombinant adenovirus with CIK cells, they were used for the treatment of an orthotopic rectal cancer mouse model. For this purpose, the mice were randomly assigned to 4 groups of CIK therapy alone, recombinant virus alone, the combination, and the control. Intratumoral infusion of the recombinant virus had better anti-tumor effects than CIK therapy alone (*P* < 0.0001). However, the combined therapy of intratumoral injection of recombinant virus and intravenous injection of CIK cells showed a significantly improved inhibition of cancer proliferation compared to the other groups (*P* < 0.001). It was hypothesized that following the use of oncolytic viruses, tumor-derived cytokines and tumor antigens are exposed, which enhances the immunogenicity of the tumor and could be beneficial for the efficacy of adoptive immunotherapy [62].

## Clinical studies on CIK Cell therapy

The reported clinical trials in the International Registry on CIK Cells (IRCC) from 1999 to 2019 demonstrated that CIK cells could be a potential safe therapy with favorable results in the treatment of cancers [63]. Many clinical studies are carried out on CRC patients to investigate the safety and efficacy of CIK cell therapy in combination with routine treatment, which is mainly chemotherapy. Zhu et al*.* assessed the therapeutic effect of CIK therapy combined with chemotherapy in a retrospective single-center study. In this study, 96 CRC patients who had undergone colectomy or proctectomy were enrolled, amongst which 21 patients were treated with CIK (1, 2, or 3 cycles) during and/or after adjuvant chemotherapy, and the other 75 patients were considered as the control group. In OS, no significant difference was observed between the two groups. The 1- and 2-year disease-free survival (DFS) rates were 89.47% and 59.64%, respectively, in the CIK group and 64.84% and 29.35%, respectively, in the control group. Moreover, no side effect to CIK cells was observed in patients. Due to the nature of retrospective studies, the study's selection bias was a significant limitation [64].

Next, in a randomized control study by Du et al*.*, patients with metastatic CRC were randomly divided into two groups; 30 patients received CIK transfusions in combination with chemotherapy (regimen of XELOX), and 30 patients were treated with chemotherapy alone. The safety and efficacy of CIK plus chemotherapy were evaluated. Although no complete remission (CR) was observed in either group and there was not a significant difference in objective response rate (ORR) between the two groups, the disease control rate (DCR) was significantly higher in the experimental group due to higher numbers of stable disease (SD) among the patients. Moreover, the median PFS was 10.15 and 8.64 months in the trial and control groups, respectively. Through assessment of the quality of life, it was demonstrated that patients receiving CIK in addition to chemotherapy had significantly improved physical function, overall health status, and fewer symptoms. Regarding side effects, CIK did not cause any severe toxicity among patients [65].

A retrospective comparative study with two sets of cohorts was carried out in the same year. Patients with resectable CRC were divided into two groups; the study group (74 patients) was treated via surgery, chemotherapy, and DC-CIK infusions, and the control group (130 patients) via surgery and chemotherapy. Although the pre-surgery and post-surgery CEA levels and tumor recurrence were not significantly different between the study and the control group, the median survival time (MST) was significantly prolonged in the immunotherapy group compared to the control group. The advanced CRC patients were divided into three groups; 11 patients were treated with chemotherapy (group C), seven patients with DC-CIK infusions (group I), and 17 patients with DC-CIK infusions and chemotherapy (group I + C). While there were no significant differences in MST between the I and I + C groups and I and C groups, MST was significantly longer in the I + C group than the C group. Regarding the side effects, aside from delayed-type hypersensitivity (DTH), fever, insomnia, anorexia, joint soreness, and skin rash, no severe toxicity was observed in patients receiving DC-CIK immunotherapy [66].

In a retrospective randomized clinical trial, to evaluate the efficacy of CIK cell therapy in combination with chemotherapy on the prognosis of CRC patients, 60 CRC patients were divided into two groups; half of the patients received chemotherapy alone, and the other half received chemotherapy in combination with CIK cell infusions. The median PFS and OS were reported to be 25.8 and 41.3 months in the CIK group compared to 12 and 30.8 months in the control group, respectively. Moreover, significant differences between the two groups were observed in the PFS and OS curves [12].

In a large-scale non-randomized retrospective study on advanced CRC patients, 100 patients received DC-CIK therapy alongside their routine treatment, and they were compared to 251 patients who were treated only with the standard treatment. The OS was significantly prolonged in the DC-CIK therapy group. The DTH skin test was performed in the study group to evaluate the immune response to DC-CIK therapy. About 62% of patients had a positive immune response. Moreover, the assessment of the quality of life demonstrated that 75.2%, 74.2%, 72.1%, and 70.1% of patients had improvement in physical strength, appetite, sleep, and body weight, respectively. No severe toxicities were observed in the study group [67].

In 2014, Gao et al*.* designed a cohort study on 54 post-surgical gastric and CRC patients to investigate OS and DFS in DC-CIK therapy. They were randomly assigned to the study and control groups; 27 patients in the control group were treated with at least one cycle of chemo-radiation. Meanwhile, 27 patients in the study group received one cycle of low dose chemotherapy, and after 2 or 3 days, were treated with one or two cycles of DC and CIK infusions according to the stage of the disease. The results demonstrated that 5-year DFS and OS were significantly prolonged in the study group, and the CRC patients of the study group were more sensitive to DC-CIK therapy [68].

A phase II clinical trial was carried out in 2016 to assess the efficacy and safety of CIK therapy in combination with chemotherapy in stage IV gastric cancer and CRC patients, in which 16 patients were enrolled as the study group and 16 as the control group, among whom six patients in each group had CRC. Following three cycles of treatment, none of the patients experienced CR. The ORR and DCR were higher in the CIK group; however, they were not statistically significant. Furthermore, the PFS and OS were also prolonged in the study group, but neither was significant. The analysis of plasma CEA, CA126, and CA19-9 showed that CA125 and CA19-9 significantly decreased after the CIK plus chemotherapy treatment. There was no significant change in the aforementioned serum tumor marker levels in the control group. No severe CIK-related toxicity was observed, and the most common side effect of CIK was fever [13].

Lin et al*.* conducted a randomized prospective study on 255 advanced CRC patients. They reported that 134 patients, who received DC-CIK therapy in combination with chemotherapy, had significant improvement in median PFS and OS. Moreover, the grade III and IV hematologic toxicities (leukopenia, anemia, and thrombocytopenia) were more frequent among patients who were only treated with chemotherapy [69].

Zhao et al*.* conducted a phase II clinical trial in 2016 to evaluate the therapeutic benefits of CIK treatment. In this study, 122 stage IV CRC patients were randomized into two groups. The study group (61 patients) received chemotherapy combined with CIK therapy, and the control group was treated with chemotherapy alone. The results indicated a significant increase in 3-year and median OS in the study group compared to the control group. However, the increases in 3-year and median PFS were not statistically significant in the study group. Moreover, the cycle count of CIK therapy significantly prolonged PFS and OS with the optimal cutoff point of 4 cycles. After univariate analysis, it was demonstrated that Karnofsky performance status (KPS) less than 80, more than one metastasis, and elevated platelets were significantly associated with a poorer prognosis in the study group. Furthermore, the assessment of clinical response showed that the ORR was higher in the study group (19% vs. 8%, respectively), but the DCR was similar between the two groups [14].

In 2017, in a randomized prospective study, 46 CRC patients were randomly assigned to the chemotherapy plus CIK therapy and chemotherapy alone groups. The recurrence rate was significantly lower in the CIK group than the control group during the first two years of follow-up. Moreover, the median survival time was significantly prolonged in the CIK group. The evaluation of the quality of life demonstrated that although the KPS score was decreased after treatment in both groups, the reduction after treatment was less in the CIK group. Levels of CEA, CA19-9, vascular endothelial growth factor (VEGF), and basic fibroblast growth factor (bFGF) were significantly reduced after both treatments. Moreover, levels of the tumor markers mentioned above were significantly lower in the CIK group in comparison with the control group after treatment (*P* < 0.05) [70].

In another retrospective study, the quality of life and prognosis of CRC patients receiving DC-CIK cells in combination with chemotherapy were compared with chemotherapy alone. In this study, 71 CRC patients were enrolled in the study group and 71 patients in the control group. Regarding the quality of life, the KPS score in the DC-CIK group was significantly higher than in the control group (76.48 vs. 67.74; *P* < 0.05). Moreover, the median survival time was 32 months in the study group. In contrast, it was reported to be 17 months in the control group, which was significantly lower than that of the DC-CIK group (*P* < 0.001). Furthermore, the DC-CIK group's 1, 3, 5-year PFS and OS rates were all significantly higher. The only observed side effects of DC-CIK infusions were fever, chills, fatigue, headache, chest tightness, and hypotension [71].

In the following year, another study was published, in which 35 CRC patients with stages II, III, IV of cancer received chemotherapy combined with DC-CIK cell transfusions, whereas the other 35 were treated with chemotherapy alone. In the study group, two patients experienced CR, 19 patients achieved partial remission (PR), and nine patients reached SD; in contrast, in the control group, one patient had CR, ten patients achieved PR, and seven patients experienced SD. The effectiveness ($\frac{CR+PR}{Total}*100$) of DC-CIK cell transfusion in combination with chemotherapy was 60%, whereas in the chemotherapy alone group was 31.43% (*P* < 0.05). After treatment, the increase in quality of life was 85.71% in the study group and 54.29% in the control group (*P* < 0.05). The adverse reactions in the study group were significantly lower than that of the control group (*P* < 0.05) [15].

Two retrospective studies of adjuvant CIK therapy in CRC patients were carried out by Pan et al*.* in 2020. In one of these studies, 60 and 62 CRC patients with varying stages received CIK therapy combined with chemotherapy and chemotherapy only, respectively, after the complete resection surgery. The DFS and OS rates significantly improved in the CIK group compared to the control group. In univariate analysis, early T stage, sufficient chemotherapy duration, and CIK therapy were significantly associated with higher DFS. Moreover, in multivariate analysis, early T stage (stages 1, 2, or 3) and CIK therapy independently improved DFS. CIK therapy was associated with significant improvement of OS in univariate and multivariate analyses. Furthermore, the subgroups analysis of T stage and chemotherapy duration demonstrated that CIK cell therapy significantly improved DFS and OS in the T4 stage group compared to the control group. In addition, in patients receiving chemotherapy for less than 20 weeks, CIK therapy significantly boosted DFS and OS. However, in low-risk stage patients and patients who received chemotherapy for more than 20 weeks, the benefit of CIK therapy was not statistically significant [23]. In the second study, a large-scale retrospective study, metastatic CRC patients were assigned to the CIK therapy group receiving CIK plus chemotherapy (126 patients) or the control group receiving only chemotherapy (126 patients). The median OS and PFS were significantly improved in the CIK group compared to the control group. Univariate and multivariate analyses showed that primary tumor of the rectum, one metastatic site, and CIK therapy significantly improved OS and PFS. In subgroup analysis, in patients with metastatic colon cancer and patients with one metastatic site, CIK therapy significantly prolonged PFS and OS. In both studies mentioned above, no severe side effects were observed among CIK therapy patients [23, 25].

In a retrospective study to analyze the clinical efficacy of DC-CIK cell therapy in CRC patients, four groups of patients were formed according to their stage of disease and treatment. Patients with CRC stage II and III were divided into the DC-CIK cell therapy plus postoperative adjuvant therapy group (16 patients) and postoperative adjuvant chemotherapy group (47 patients), while patients with stage IV CRC received either palliative care in combination with DC-CIK cell therapy or palliative care alone. The 5-year DFS rate and DFS were significantly better in the postoperative adjuvant chemotherapy plus DC-CIK cell therapy group (*P* < 0.05). However, the median OS and survival rates were not statistically significant between two groups of stage IV CRC patients [16].

In Table 1, the details of the aforementioned clinical studies are illustrated. Altogether, 1,969 CRC patients were enrolled in 14 clinical trials, of which 842 patients received CIK cells in combination with routine therapy with or without DCs. Nine studies reported the median follow-up time, ranging from 10.5 to 54.5 months. Among studies on exclusively metastatic CRC patients, significantly improved OS after treatment with CIK cells was reported in a total of four studies, including 421 patients in study groups and 559 patients in control groups; meanwhile, significantly improved PFS was reported in three studies, including 290 patients in study groups and 277 patients in control groups. Among all the clinical trials in the present review, eight studies, including 595 patients in treatment groups and 735 patients in control groups, demonstrated significantly improved OS. In addition, five studies, including 391 patients in treatment groups and 378 patients in control groups, reported significantly improved PFS after CIK cell therapy. The findings indicate that CRC patients who are candidates for adjuvant chemotherapy and radiotherapy (high-risk stage II, stage III, and stage IV) could be the target group for CIK cell therapy. Based on three trials reporting the clinical response to CIK therapy, the ORR and DCR were 42/119 and 103/119, respectively.

**Table 1:** Clinical studies on CIK cell therapy combined with chemotherapy

Author	Year	Stage of CRC	Method	Median follow-up period (months)	Sample size		Intervention		Efficacy	Safety
					Study	Control	Study	Control		
Zhu [64]	2013	Variable	Retrospective single-center study	19	21	75	9*10^9^ CIK cells per cycle which was transfused during and/or after chemotherapy. During chemotherapy there was a 3 days interval between CIK and chemotherapy. CIK cycles were at least 4 weeks apart	Variable chemotherapy regimens (Oxaliplatin + Fluorouracil, or Irinotecan + 5-FU, or 5-FU, or Other)	There was no significant difference between two groups in OS. The DFS was statistically prolonged in the CIK group	No immediate reaction to CIK cell transfusion
Du [65]	2013	IV	Randomized control study	10.5	30	30	XELOX chemotherapy + 5*10^9^ CIK cells in one hour every other day for 3 times after each cycle of chemotherapy	XELOX chemotherapy	ORRs (CR + PR) were 12/30 and 11/30 in the study and control group, respectively (*P* = 0.791). The DCRs (CR + PR + SD) were 26/30 and 17/30 in the study and control group, respectively, which were significantly different (*P* = 0.010) PFSs were 10.15 and 8.64 months in the study and control group, respectively	Temporary fever, mild to moderate fatigue, and joint pain in the study group
Niu [66]	2014	II, III	Retrospective comparative study	16.3	74	130	IV infusion of 1*10^7^ DC cells on days 8, 15, and 22 and intradermal injection on days 29, 36, and 43 + IV infusion of 1*10^9^ CIK cells on days 11 to 14 + Capecitabine; or Capecitabine plus Oxaliplatin; or Oxaliplatin, Leucovorin, and 5-FU	Capecitabine; or Capecitabine plus Oxaliplatin; or Oxaliplatin, Leucovorin, and 5-FU	MSTs in the study and control groups were 198 and 106 days, respectively, which was longer in the study group (*P* = 0.02)	DTH, fever, insomnia, anorexia, joint soreness, skin rash were observed in DC-CIK groups
		IV		NA	7 (group I) / 17 (group I + C)	11 (group C)			MSTs in the I, I + C, and C groups were 249, 264, and 110 days, respectively. The only significant difference was between C and I + C groups (*P* = 0.04)	
Zhang [12]	2014	Variable	Retrospective randomized clinical trial	NA	30	30	6 cycles of chemotherapy regimen (FOLFOX or XELOX), + 1–4 cycles of CIK cell therapy with a one-month interval with the chemotherapy	6 cycles of chemotherapy regimen (FOLFOX or XELOX)	The median PFS and OS were improved in the CIK group (25.8 and 41.3 months vs. 12 and 30.8 months, respectively). The PFS and OS curves indicated that there were significant differences between two groups in PFS (*P* = 0.01) and OS (*P* = 0.037)	NA
Zhu [67]	2014	IV	Open-label, non-randomized, retrospective study	16.3	100	251	1*10^7^ DC cells from day 8 (first 3 weeks IV and last 3 weeks intradermally), and daily IV infusion of 1*10^9^ CIK cells from day 11 for four days + routine treatment	Routine treatment	OS was significantly prolonged in the study group (*P* = 0.04)	No severe toxicities were observed in the DC-CIK group. The adverse reactions were fever, insomnia, anorexia, joint soreness, and skin rash
Gao [68]	2014	Variable	Cohort	NA	27 (13 CRC patients)	27 (13 CRC patients)	One or two cycles with an average of 188 ± 79*10^6^ DC cells and 58.8 ± 22.3*10^8^ CIK cells per cycle were infused 2 or 3 days after low dose chemotherapy	At least one cycle of chemo-radiation	5-year DFS rates of CRC patients in the study and control groups were 66% and 8%, respectively. OS rates of CRC patients in the study and control groups were 75% and 15%, respectively. OS and DFS were prolonged significantly in the DC-CIK therapy group (*P* < 0.01)	Fever was the most common side effect of DC-CIK therapy
Xu [13]	2016	IV	Phase II clinical trial	12	16 (6 CRC patients)	17 (6 CRC patients)	CIK cell infusions on days 14 and 16 of the first and second chemotherapy cycles + Chemotherapy with Capecitabine and Oxaliplatin (CAPOX)	Chemotherapy with XELOX	The ORR and DCR were higher in the study group (ORR: 25% vs. 5.9%; DCR: 62.5% vs. 58.8%). The median PFS and OS were prolonged in the CIK group (5.6 and 13.9 months vs. 3.83 and 11 months, respectively), however, they were not significant (PFS: *P* = 0.06; OS: *P* = 0.27)	No severe CIK-related toxicity was observed. The most common side effect of CIK was fever
Lin [69]	2016	IV	Randomized prospective study	NA	134	121	5 cycles of in total 1*10^7^ DCs and 1*10^9^ CIK cell treatment at one-month interval + 6 cycles of chemotherapy regimen (5-FU, FOLFOX, or XELOX)	6 cycles of chemotherapy regimen (5-FU, FOLFOX, or XELOX)	The median PFS and OS were improved in the CIK group (8.8 and 14.7 months vs. 5.8 and 10.8 months, respectively)	Grade III and IV leukopenia, anemia, and thrombocytopenia were significantly more frequent in the control group
Zhao [14]	2016	IV	Phase II clinical trial	27	61	61	5*10^9^ CIK cell infusion on days 15 and 16 per cycle with 2-week intervals with chemotherapy + 12 cycles of chemotherapy with FOLFOX4	12 cycles of chemotherapy with FOLFOX4	The 3-year and median OS were significantly higher in the study group in comparison to the control group (48% vs. 23%, *P* < 0.01; and 36 months vs. 16 months, *P* < 0.01, respectively). There was no significant difference in the 3-year PFS rate between the two groups, but the median PFS in the study group was superior to that in the control group (*P* = 0.072). The ORR was higher in the study group (19% vs. 8%; *P* = 0.049), but the DCR was similar between the two groups (89% vs. 84%; *P* = 0.491)	No grade III or IV severe side effects with CIK cell therapy
Peng [70]	2017	II, III	Randomized prospective study	NA	23	23	Two cycles of 2*10^9^ and 6*10^9^ CIK cell infusion, once a day for 3 days + chemotherapy with FOLFOX4	chemotherapy with FOLFOX4	The MST was significantly prolonged in the study group (41.9 vs. 33.8 months; *P* < 0.05). The recurrence rate during the first 2 years of follow-up was significantly lower in the study group (26.1% vs. 43.5%; *P* < 0.05)	Two cases of the CIK group developed a fever. The side effects were significantly lower in the CIK group
Xie [71]	2017	III, IV	Retrospective	76	71	71	At least two cycles of 1–2*10^10^ DC-CIK cells during 2-week-intervals of chemotherapy consisting of 4 infusions administered twice a day for 4 days + Chemotherapy with FOLFIRI every two weeks	Chemotherapy with FOLFIRI every two weeks	MST was prolonged in the study group (32 vs. 17 months; *P* < 0.001). 1, 3, and 5-year PFS rates were 85.3, 64.1, 57.4%, respectively, in the study group, which were higher than 65.0, 44.3, 33.6%, respectively, in the control group (*P* = 0.017). 1, 3, 5-year OS were also higher in the study group (*P* = 0.001)	6 patients receiving DC-CIK developed mild fever, chills, and fatigue; and three and one patients experienced headache and chest tightness with hypotension, respectively
Liu [15]	2019	II, III, IV	Cohort study	NA	35	35	4 courses of 5*10^9^ DC-CIK cells from day 14 to 16 + 4 to 6 cycles of chemotherapy with XELOX from day 1 to 14 (duration of a cycle: 21 days)	4 to 6 cycles of chemotherapy with XELOX from day 1 to 14 (duration of a cycle: 21 days)	The ORRs in the study and control groups are 60% and 31%, respectively. The effectiveness ($\frac{CR+PR}{Total}*100$) was significantly higher in the study group (*P* < 0.05)	The side effects were significantly lower in the study group
Pan [23]	2020	II, III, IV	Retrospective study	54.5	60	62	Complete resection + 4 cycles of CIK cell immunotherapy 4 weeks after chemotherapy with 1-week interval and the 4 cycles with 2-weeks interval + adjuvant chemotherapy (FOLFOX or XELOX or single-agent Capecitanine regimen)	Complete resection + adjuvant chemotherapy (FOLFOX or XELOX or single-agent Capecitanine regimen)	The DFS and OS rate significantly improved in the CIK group as compared to the control group (*P* = 0.0024 and *P* = 0.008, respectively)	Among CIK receiving patients, only 10 patients developed side effects (fever, transient hypertension, pruritus, fatigue)
Pan [25]	2020	IV	Retrospective study	55.2	126	126	12*10^10^ CIK cell infusions every 2 or 3 weeks in intervals of chemotherapy + Chemotherapy with FOLFOX, FOLFIRI, or XELOX regimen, with or without bevacizumab or cetuximab	Chemotherapy with FOLFOX, FOLFIRI, or XELOX regimen, with or without bevacizumab or cetuximab	The median OS was significantly higher in the CIK group as compared to the control group (54.7 vs 24.1 months; *P* < 0.0001). The median PFS was also significantly improved in the CIK group (25.7 vs. 14.6 months; *P* < 0.0001)	Among patients receiving CIK therapy, only 14 patients experienced mild adverse events (fever, transient hypertension, fatigue, anorexia)
Xu [16]	2020	II, III	Retrospective study	NA	16	47	At least 2 cycles of two infusions of over 5*10^9^ for DC-CIK cells, two days after chemotherapy + At least 2 cycles of postoperative adjuvant chemotherapy for 6 months	At least 2 cycles of postoperative adjuvant chemotherapy for 6 months	The 5-year DFS rate was significantly better in the study group as compared to the control group. (25% vs. 4.25%; *P* < 0.05). However, the improvement of 1 and 2-year DFS rates was not significant in the study group as compared to the control group. DFS was significantly longer in the study group (42.4 vs. 23.5 months; *P* < 0.05)	No serious adverse reaction was observed
		IV			18	35	At least 2 cycles of two infusions of over 5*10^9^ for DC-CIK cells + Chemotherapy regimen (FOLFOX, XELOX or FOLFIRI) ± Targeted therapy	A chemotherapy regimen (FOLFOX, XELOX, or FOLFIRI) ± Targeted therapy	The differences between the two groups in 1, 3, and 5-year survival rates and median OS were not significant	

*5-FU* fluorouracil; *OS* overall survival; *DFS* disease-free survival; *XELOX* capecitabine plus oxaliplatin; *ORR* objective response rate; *CR* complete remission; *PR* partial remission; *DCR* disease control rate; *SD* stable disease; *PFS* progression-free survival; *MST* median survival time; *DTH* delayed-type hypersensitivity; *FOLFOX* leucovorin, fluorouracil, and oxaliplatin; *NA* not available; *FOLFOX4* 5-FU, leucovorin, and oxaliplatin; *FOLFIRI* irinotecan, leucovorin, 5-FU

## Conclusion

Due to the limitations of routine treatments, including chemotherapy and radiotherapy in advanced CRC patients, immunotherapy could be a turning point in prolonging survival in CRC patients. A high number of studies have evaluated the safety and clinical efficacy of CIK therapy in CRC patients. While the results are variable, they demonstrate that CIK therapy could be a potential option for future CRC treatment regimens. One of the obstacles of CIK therapy is the need for a high number of CIK cell transfusions due to its limited migration ability. However, different methods, including CEA-specific CAR-CIK cells or modification of chemokine receptors of CIK cells, are being investigated to overcome this limitation. The signaling pathways involved in the CIK therapy are not yet completely apparent, whereas the knowledge could guide the strategies needed for optimal use of CIK therapy.

## Data Availability

Not applicable.
